# Reference Levels for Diagnostic and Interventional X-ray Exposures in Germany: Development, Handling, and Perspective

**DOI:** 10.1055/a-2790-4742

**Published:** 2026-03-16

**Authors:** Hugo de las Heras Gala, Augusto Giussani, Helmut Schlattl, Sebastian Trinkl, Gunnar Brix

**Affiliations:** 1 External and Internal Dosimetry, Biokinetics 84634 Federal Office for Radiation ProtectionNeuherbergBayernGermany; 2 39419 Federal Office for Radiation ProtectionGermany, retired

**Keywords:** x-ray exposures, radiation protection, diagnostic reference levels (DRLs), diagnostic radiology, national regulation, optimization

## Abstract

**Purpose:**

Diagnostic reference levels (DRLs) serve to optimize diagnostic and interventional X-ray applications, which account for over 95% of man-made radiation exposure in Germany. The national DRLs are regularly adjusted to reflect advances in device technology and clinical practice. The aim of this article is to present the basic concept of DRLs, their methodological development, and the resulting improvements in patient protection.

**Materials and methods:**

The relevant regulations of the new radiation protection legislation in Germany are summarized, the development of DRLs between 2003 and 2022 is described, guidance for the practical application of DRLs and the implementation of supplementary facility-specific measures is provided, and finally an outlook on future issues is given.

**Results:**

Since the first official announcement of DRLs in Germany, they have been reduced remarkably through continuous protocol optimization and the use of more advanced hardware and software. Over the period of review of almost 20 years, the catalog of X-ray exposures for which DRLs have been established has been expanded considerably, although some DRLs have been removed for various exposures that are no longer routinely indicated.

**Conclusion:**

The consistent implementation of the DRL concept has proven effective in practice and has led to a substantial reduction in patient exposure over the years. This positive development in terms of patient protection justifies the personnel effort as well as the potential need to update hardware and software. Despite the success achieved, the DRL concept requires continuous further development in the future.

**Key Points:**

- Diagnostic reference levels (DRLs) are an effective tool to optimize X-ray exposures.
- In Germany, many DRLs have been more than halved in nearly 20 years.
- With good equipment and practice, DRLs can and should be considerably undercut.
- Dose management systems considerably facilitate the practical implementation of the DRL concept.
- The national DLRs should be complemented by facility-specific local DRLs.

**Citation Format:**

- de las Heras Gala H, Giussani A, Schlattl H et al. Reference Levels for Diagnostic and Interventional X-ray Exposures in Germany: Development, Handling, and Perspective. Rofo 2026; DOI 10.1055/a-2790-4742

## Introduction

In Germany, diagnostic and interventional X-ray applications are responsible for over 95% of the population's exposure to man-made radiation sources. So, it is important to ensure that the two main principles of medical radiation protection – justification and optimization – are carried out on a consistent basis. This applies particularly to higher-dose applications, such as CT scans and angiographies or vascular interventions, which in 2021 accounted for approximately 71% and 14%, respectively, of the population’s medical radiation exposure from X-ray applications, although these procedures only represented approximately 11% and 3%, respectively, of the total frequency of medical X-ray applications 1 2 .

A key element for promoting optimization is the concept of diagnostic reference levels or DRLs that was initially proposed by the International Commission on Radiological Protection (ICRP) and has since become law in the European Union and Germany. DRLs are defined for frequent and/or higher-dose X-ray applications. These levels should be followed when using standard equipment for a group of patients, but they are not threshold values. With state-of-the-art equipment, these values can and should be considerably undercut. When current DRLs are not being met, operators will need to carefully optimize the protocols they use and, in extreme cases, modernize their devices. The DRLs have to be updated at regular intervals based on representative data collections, in order to take account, for example, of the rapid advances in equipment technology. As part of regular updates, operators also need to check whether the DRLs for applications no longer routinely indicated – due to new equipment technology or new types of examination – can be eliminated or whether new DRLs need to be defined. A compilation of the currently valid DRLs and (for informational purposes) the corresponding effective dose values is provided as **supplementary material in Tables S1-S10** .

To summarize, this review article:

- addresses changes in the concept of DRLs as well as corresponding updates to the German Radiation Protection Act;
- highlights the successes achieved over the years in terms of reducing patient exposure;
- provides guidance on applying some of the current DRLs in practice;
- proposes facility-specific quality assurance measures for X-ray applications that support and expand implementation of the DRL concept nationwide.

## ICRP recommendations for the DRL concept and its legal and practical implementation in Germany

The concept of DRL was first introduced by the ICRP in 1996 3 , and it was further refined and developed in several subsequent publications 4 5 6 . The most recent publication recommends using median values to characterize the dose distributions collected by operators on the basis of a sample (see below), because this approach does not require any assumptions about the distribution of sampled values. The arithmetic mean, on the other hand, which has been used more frequently in practice, provides only a meaningful estimate of expected values for standard distributions. In addition, the median value is affected less by outliers.

In Europe, the concept of DRLs was first introduced in 1997 in the form of the 97/43/EURATOM guidelines 7 , and it was refined and consolidated in the 2013/59/EURATOM guidelines 8 . In Germany, the concept was introduced in the Radiation Protection Ordinance of 2001 (Sec. 81, No. 2) 9 and the X-ray Ordinance of 2003 (Sec. 16, No. 1) 10 , and it was extended in the new Radiation Protection Act (StrlSchG) 11 , which came into force on December 31, 2018, and in the associated new Radiation Protection Ordinance (StrlSchV) 12 . From a terminology perspective, it should be noted that in German legislation diagnostic and interventional X-ray applications are grouped under the general term “examinations”.

As illustrated in Fig. 1 , three main stakeholders are involved in implementing the concept in Germany: the Federal Office for Radiation Protection (BfS), the operators of medical X-ray devices, and the medical or dental bodies.

**Fig. 1:**
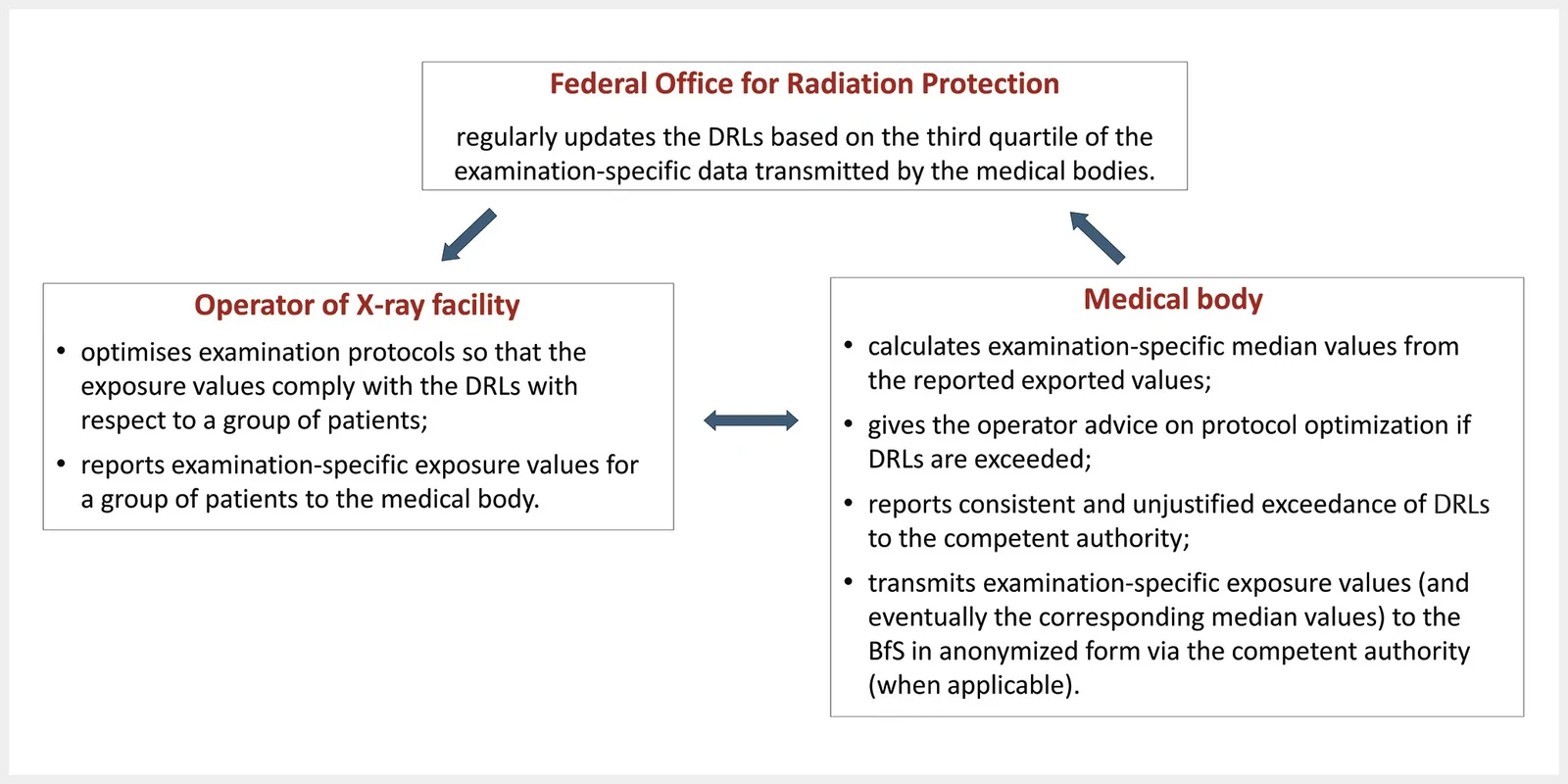
Schematic representation of the cyclic DRL concept implemented in Germany. The main tasks of the three involved parties are briefly mentioned; further details are explained in the text.

According to Sec. 122 Para. 3 StrlSchV, operators (represented by the respective radiation protection executive, i.e., the person with overall responsibility for radiation protection) must take the DRLs published by the BfS as a basis in examinations of persons. As mentioned, these levels do not represent thresholds for examinations of individuals but instead refer to a group of patients undergoing the respective examination.

Compliance with the DRLs must be checked by the responsible medical and dental bodies. For this purpose, as part of their routine checks for different types of examinations, operators must request the corresponding operational exposure parameters for at least 10 examinations 1 , calculate the associated median values, and compare them with the respective DRLs, as described in the “Guidelines for Handling Diagnostic Reference Levels for Diagnostic and Interventional X-ray Applications” 13 .

It should be noted that the method of sample collection is not uniformly specified by the medical bodies. Some medical bodies request exposure data for X-ray applications that were either provided for the operators in the X-ray logbook or that were carried out within a specified period of time. Others request data for a specific number of consecutively performed X-ray applications. However, there are also medical bodies that forgot specific requirements entirely and allow the operators to select the exposure data to be transmitted. The latter carries the risk that deliberately low exposure levels will be reported.

In the event that the values persistently and unjustifiably exceed the DRLs, the medical or dental body reports this to the responsible authority (Sec. 130 Para. 3 Sentence 1 No. 3 StrlSchV). It should be emphasized that compliance with the DRLs does not replace the optimization requirement according to Sec. 83 Para. 5 StrlSchG. The DRLs are not optimal or target values, but rather they can and should be undercut if the image quality achieved is sufficient for diagnostic evaluation. If there is potential for operators to optimize their values, the medical or dental bodies will advise them accordingly.

The DRLs have to be updated regularly to keep up with the latest advances in technology and medical sciences. According to Sec. 125 Para. 1 Sentence 3 StrlSchV, the values of patient exposures collected by the medical and dental bodies must be transmitted to the BfS annually in anonymized form. According to Sec. 125 Para. 2 StrlSchV, the BfS checks at least every three years whether the DRLs need to be adjusted, and then they updates, as necessary. The determination of the revised DRLs is based on the 3rd quartile of the median values of the data transmitted by the medical or dental bodies. For the initial determination of new DRLs for which no data from the medical or dental bodies are available yet, operational dose values from other national sources and surveys are used 14 .

Due to the method of data sampling and processing specified, the statistical parameters – contrary to a frequently expressed opinion – do not refer to the ICRP’s relatively slim reference person but rather to typical patients. Based on this approach, a comprehensive consultation process takes place with representatives from all of the relevant stakeholders, in order to ensure that all clinical aspects related to an application are taken into consideration. After weighing all of the aspects discussed, the BfS determines the new and revised values of the DRLs and publishes them in the Federal Gazette.

## Development of DRLs over time in Germany

National DRLs for X-ray applications were first published in Germany in 2003 15 , and they have been updated to reflect the latest advances in radiological technology and clinical practice in 2010 16 , 2016 17 , 2018, 18 and 2022 19 . On the one hand, a range of new DRLs has been added (including values for dental and maxillofacial digital volume tomography (DVT/CBCT), as well as tomosynthesis of the breast). On the other hand, DRLs for examinations that no longer match current practices and are only indicated in special cases have been removed from the catalog (including pediatric X-rays of the skull). Currently, there are a total of 52 DRLs for diagnostic and interventional X-ray applications for adults (2003: 25) and seven for children and adolescents (2003: 7), the latter of which are divided into as many as six weight/age groups.

The development of the DRLs over the years can be clearly traced based on the examinations for which the DRLs were updated at least three times ( Fig. 2 ) for adults after their initial publication in 2003 and at least twice ( Fig. 3 ) for children/adolescents after initial publication in 2003 or 2010. For the purpose of comparison, the corresponding European DRLs 20 21 22 are shown, insofar as data are available and comparable.

**Fig. 2:**
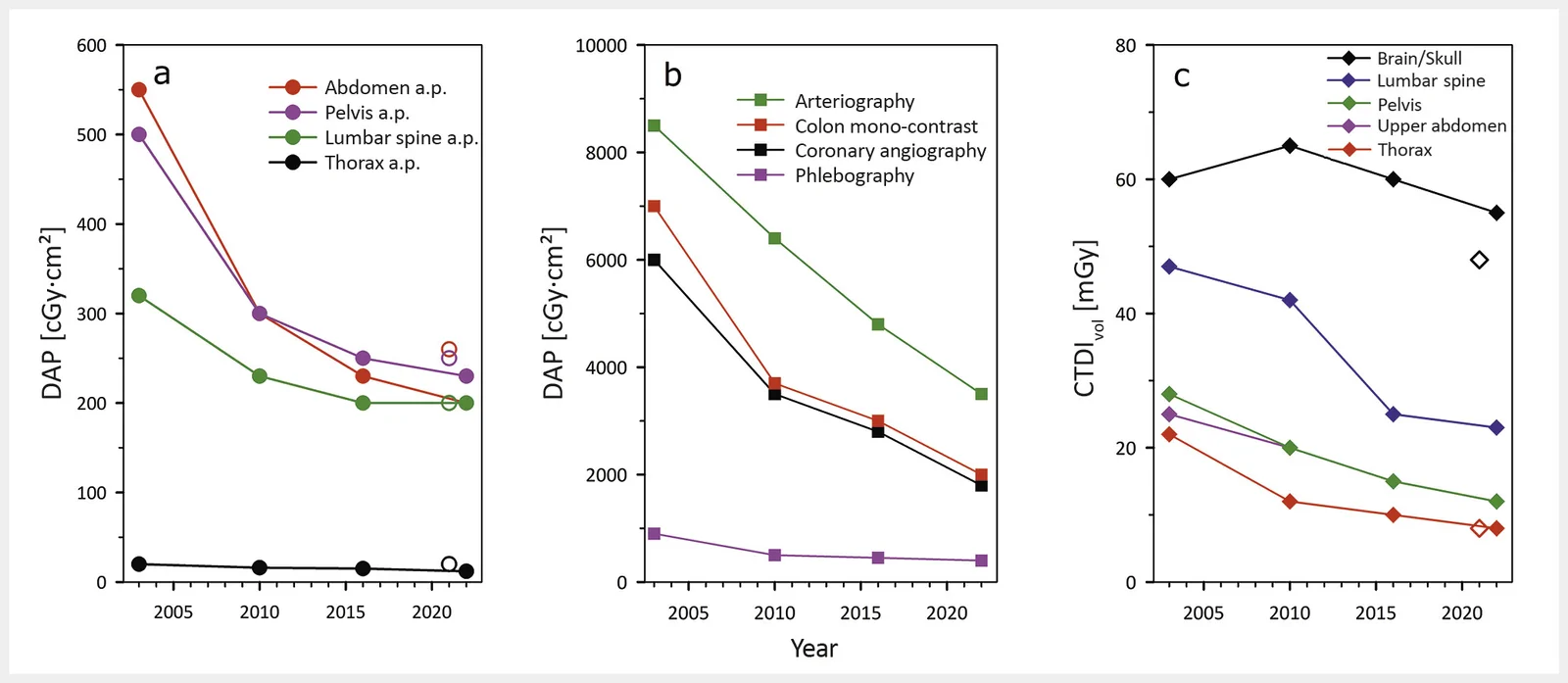
Temporal development of diagnostic reference levels (DRLs) in Germany (filled colored symbols) for comparable X-ray applications in adults, for which four DRLs have been established between 2003 and 2022. **a** Planar X-ray imaging, **b** fluoroscopy and interventions, and **c** computed tomography. For comparison, corresponding European DRLs 20 21 are shown (open colored symbols). Note: The CTDI _vol_ values established in 2003 took into account that both scans are acquired in some examinations. To enable a fair comparison with the DRL values set in the subsequent updates, the 2003 values were thus converted to relate to a single scan.

**Fig. 3:**
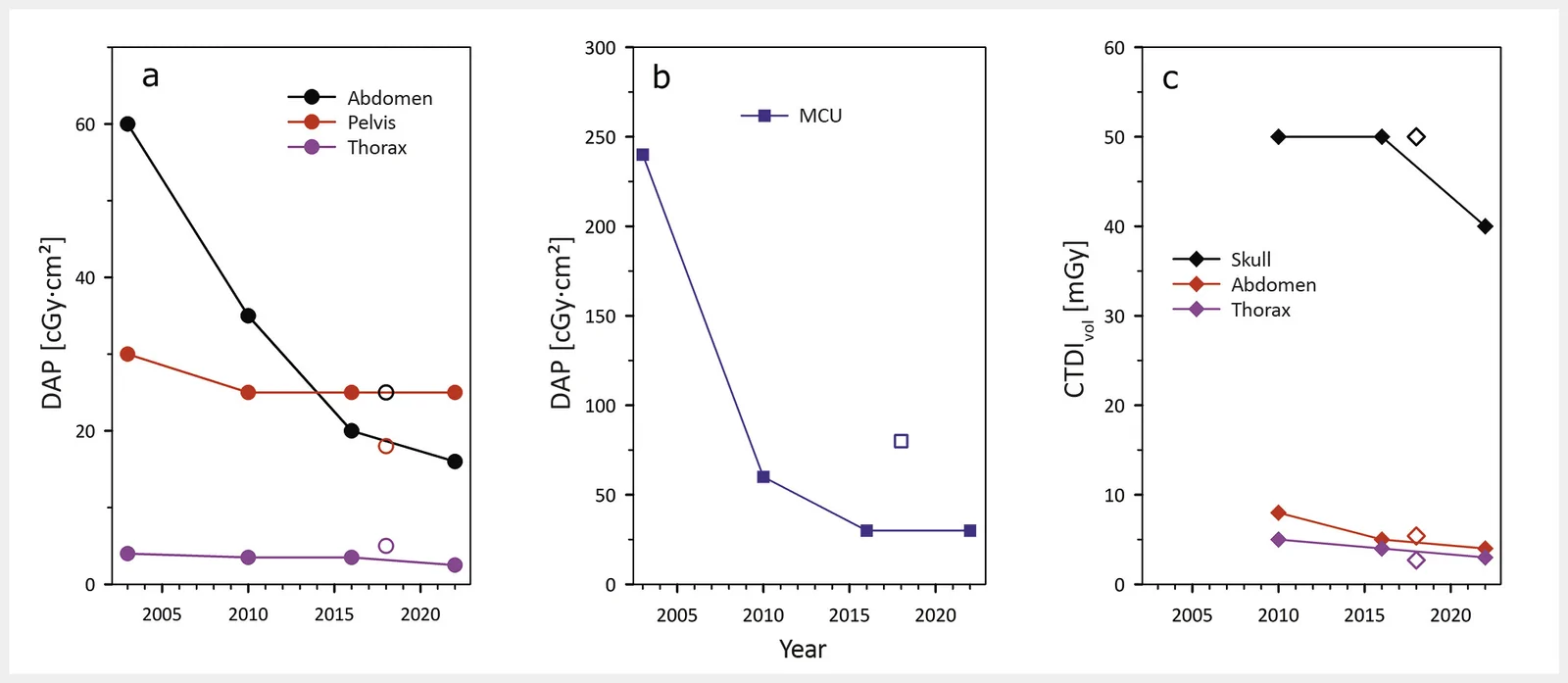
Temporal development of DRLs in Germany (filled colored symbols) for comparable X-ray exposures in children, for which at least three DRLs have been established between 2003/2010 and 2022. Data are presented as examples for the weight group “middle childhood” (19 to 32 kg, approx. 5 to 10 years old). **a** Planar X-ray imaging, **b** micturating cystourethrography (MCU) and **c** computed tomography. For comparison, corresponding European DRLs from 2018 22 are shown (open colored symbols).

As the figures illustrate, a substantial reduction in DRLs has been observed over the years for the vast majority of applications; this trend was usually quite pronounced in the beginning and then leveled off somewhat. The marked reduction at the outset can be attributed to two factors: (i) When the DRLs were first defined, the database available was still insufficient and not representative, so that in some cases the DRLs set were not too restrictive. (ii) With the introduction of DRLs, many operators recognized the potential for reducing patient exposures by consistently optimizing their protocols; subsequent reductions, on the other hand, have been driven primarily by ongoing updates to hardware and software in the market. Due to rapid advances in technology in the field of medical imaging, the latter trend is expected to continue.

## Conceptual changes in the most recent update (2022)

Based on experience gained, the definition of DRLs has undergone various modifications in Germany over the years. The main changes in the latest update addressed the following aspects:

- For CT scans, DRLs were only determined based on the specified volume CT dose index ( *CTDI*_vol_ ) and no longer on the dose length product ( *DLP* ) as the primary quantity. The background to this is, that, unlike the CTDI _vol_ , the DLP depends strongly on the scan length and thus on the clinical question and the resulting justifying indication. This is also one of the reasons, for example, that it has been possible to establish uniform DRLs for CT examinations of the spine (cervical, thoracic, and lumbar spine). Nevertheless, for the purposes of guidance, typical scan lengths for the respective examinations were also published. However, it is recommended to maintain facility-specific alarm thresholds based on the *DLP* .
- Prospectively triggered and retrospectively ECG-synchronized coronary angiographies were assigned standardized DRLs, where retrospectively synchronized examinations should only be performed under special circumstances (e.g. in patients with arrhythmias).
- DRLs for endovascular aortic repair (EVAR) were established taking into account their complexity, with one value each for simple and complex/branched prostheses.
- Separate DRLs were also established for implantation of pacemakers or defibrillators, based on the complexity of the procedures, i.e. the number of chambers involved.

## Instructions for use of DRLs

The following section provides more detailed explanations regarding the use of various DRLs in practice.

- *CT scans of multiple body regions* : When examining multiple body regions, separate scans should be performed whenever possible, each adhering to the DRLs defined for the respective body region/indication (complying with guidelines from the German Medical Association 23 ). If combining several body parts in one scan is clinically indicated (e.g. accident patients), then the *CTDI*_vol_ for the body part with the highest DRL must be used.
- *DRLs for dental CBCT:* In the case of particularly large volumes that cover more than the oral cavity, not enough data is available currently to set a DRL. In this case, the DRL for PNS studies of 1,500 mGy cm² can be used. However, large volumes should be the exception in dentistry and must have a specific justifiable indication.
- *DRLs for interventions* : An intervention according to Sec. 1 Para. 8 StrlSchV includes both diagnostic and therapeutic purposes. If the diagnostic component can be performed independently from the therapeutic component, then the dose area product (DAP) for this application should be compared with the respective DRL. However, if the intervention includes a therapeutic component in addition to a diagnostic one, the entire procedure is considered therapeutic, and the corresponding DRL for operational procedures must be observed. If the dose during an intervention is expected to exceed or has exceeded the relevant DRL due to the complexity of the procedure, a corresponding justification must be documented, e.g. by stating the number of vessels affected.
- *Age groups in pediatrics* : The 56-kg age group (starting at approx. 15 years of age) corresponds to the size of a slim adult, so no separate DRLs for young people have been set for this weight class.
- *Planning CT in radiotherapy:* Although these special applications are not explicitly included in the DRL catalog, in order to avoid any unnecessary exposure during CT scans for radiation planning (“planning CT”), operators should observe the DRL established for diagnostic CT scans of the respective body region. This follows from the ALARA principle for protecting healthy tissue, even if the exposure in the target volume resulting from the subsequent radiotherapy is substantially higher than that of the planning CT scan.
- *Auxiliary CT scans (low-dose CT) for attenuation correction and/or anatomical co-registration in hybrid studies using PET/CT and SPECT/CT systems:* For these studies, operators should apply the DRL published for nuclear medicine examinations 24 . However, if images of diagnostic quality are acquired, operators should use the relevant DRLs currently published for X-ray applications.

## Recommendations for facility-specific measures to implement and extend the DRL concept

The DRLs listed in BfS publications refer to frequent and/or high-dose X-ray applications. They include neither concrete recommendations for optimizing countless other X-ray applications nor practical tips for the operators. More detailed recommendations are provided in the following:

- *Dose management systems* . It is strongly recommended that dose-relevant parameters for all X-ray devices in a facility be recorded continuously and automatically using a dose management system 25 26 27 and evaluated systematically as part of the facility’s quality management system. Information on the patient's body size (e.g. height, weight, body circumference, BMI) should also be taken into account, if possible, as this information could help to justify exposures that exceed specified DRLs during inspections by the medical or dental bodies.
- *Local reference values.* Despite substantially expanding the DRL catalog, there are still many X-ray applications for which no national DRLs have been established, either because they are not particularly dose-intensive or because they are not used very frequently across the country. However, if such examinations are carried out more frequently in a facility, it is recommended to establish local DRLs based on the 3 ^rd^ quartile of a larger local patient population 28 29 30 31 32 . It is also recommended to set local DRLs for types of examinations where national DRLs are established but can be undercut significantly by using state-of-the-art devices and good practices. These local DRLs could, for example, be based on the median values of the national distributions of the corresponding dose-relevant parameters, which can be found in the Supplement.
- *Local quality assurance teams* . To optimize patient radiation protection – going beyond compliance with the DRL – it is strongly recommended to establish local teams consisting, for example, of physicians, medical physicists, IT managers, and medical technologists for radiology, who regularly analyze and evaluate examination-specific patient exposures. The aim should be to optimize patient exposures even below the DRL, in order to achieve the required image quality with the lowest possible exposure in accordance with Sec. 14 Para. 1 No. 5a StrlSchG and Sec. 130 StrlSchV. Special attention should be given to X-ray applications that have very broad distributions of operational dose parameters ( *DAP* , *CTDI*_vol_ or *DLP* ).

## Conclusions and outlook

The DRL concept has been implemented consistently in practice over the years, and its success, thanks to efforts to consistently optimize protocols and upgrade to state-of-the-art equipment, has led to significant reductions in patient exposure for X-ray applications in line with the ALARA principle. This positive development in terms of patient protection justifies the personnel costs and the upgrades required for hardware and software. Despite many successes achieved, further consideration is being given to developing both the DRL catalog and the basic concept.

In order to prevent a systematic bias in the exposure data collected from operators by medical or dental bodies, the procedure for sampling the data needs to be standardized, at least to the degree that the choice of X-ray applications is not left to the operators but instead follows specific requirements set by the medical or dental bodies. This is the only way to avoid that operators report below-average exposure values, which are then forwarded in anonymized form to the BfS and used for the next update of the DRLs.

DRLs have not yet been established for some clinically relevant X-ray applications due to a lack of representative data. This applies, for example, to CT perfusion scans of the brain or heart. For guidance in this regard, operators should refer to the considerations and exposure levels provided by Brix et al. 33 . Additionally, a research project by Borowski et al. 34 has demonstrated that DRLs for CBCT applications in the trunk area cannot be derived, as hoped, in a meaningful way from the corresponding values for CT examinations. Representative surveys are thus urgently needed in this regard.

It is also possible that operators may reduce exposure parameters to comply with DRLs to such an extent that, due to outdated devices and software, the image quality is no longer sufficient for reliable diagnosis. This can currently only be avoided if the medical or dental bodies evaluate compliance with the DRLs in conjunction with the image quality achieved, which is not always done. In the future, it would be conceivable to expand the DRL concept with an image quality reference value that would refer to one or more parameters characterizing image quality (e.g. the noise level or the detectability of well-defined image features). These parameters could initially be automatically calculated, documented, and correlated with a medical assessment of the diagnostic image quality after each examination using the acquired or reconstructed image data. First forays in this direction have already been published 35 36 37 38 39 . Conceptually, it would make sense to combine these approaches with AI technologies such as machine learning.

## Supplementary Material

Supplementary Material Zusatzmaterial

Supplementary Material Zusatzmaterial

## Zusatzmaterial

Supplementary Material Zusatzmaterial

Supplementary Material Zusatzmaterial

## Footnotes

1 In order to collect data for updating purposes, also less than 10 values may be transmitted to the medical or dental bodies and, subsequently, by them to the BfS for paediatric or otherwise rarely performed types of examination.
