# Supplementary material for: Reference Levels for Diagnostic and Interventional X-ray Exposures in Germany: Development, Handling, and Perspective
Source: Rofo. 2026 Mar 16;198(11):1444–51. doi: 10.1055/a-2790-4742 (PMC13716514; doi:10.1055/a-2790-4742)
Supplement: Supplementary file 1 [file 10-1055-a-2790-4742_28410570.pdf]

Supplement

Zusammenstellung der 2022 bekanntgemachten diagnostischen Referenzwerte (DRW) mit den entsprechenden Werten der effektiven Dosis sowie zur Orientierung die Medianwerte der bundesweit bei Betreibern erhobenen Expositionswerte

Tabelle S1: DRW für konventionelle Projektionsaufnahmen am Erwachsenen

Untersuchungsart	*DFP* [a] [cGy·cm^2 = µGy·m^2]		Effektive Dosis [mSv]
	Medianwert	*DRW*	
Schulter (pro Ebene) [b]	13	25	0,009
Thorax p.a. [c]	8	12	0,030
Thorax a.p. [c] (im Liegen)	9	15	0,075
Thorax lat. [c]	22	40	0,11
Brustwirbelsäule a.p./p.a. [d]	60	100	0,43
Brustwirbelsäule lat.	65	120	0,17
Lendenwirbelsäule a.p./p.a. [d]	122	200	0,54
Lendenwirbelsäule lat.	200	330	0,34
Abdomen a.p./p.a. [d]	123	200	0,51
Becken a.p./p.a. [d]	145	230	0,36
Hüfte (pro Ebene)	57	100	0,093

[a] *DFP*: Dosis-Flächen-Produkt
[b] Bei Untersuchungen in 2 Ebenen liegt der DRW bei 50 cGy cm^2, auch wenn eine Y-Aufnahme inkludiert ist.
[c] a. p.: anterior-posterior; p. a.: posterior-anterior; lat.: lateral
[d] Laut Bundesärztekammer-Leitlinie (https://doi.org/10.3238/arztebl.2023.LL_Qualitaetssicherung_Roentgen-diagnostik) sind nur a.p.-Aufnahmen empfohlen.

Tabelle S2: DRW für mammographische Untersuchungen

Untersuchungsart	*AGD* [a] [mGy]		Effektive Dosis [b] [mSv]
	Medianwert	*DRW*	
Mammographie (pro Ebene)	1,4	2,0	0,28
Tomosynthese (pro Ebene)	1,9	2,5	0,34

[a] AGD: Average glandular dose (mittlere Parenchymdosis)
[b] Die Werte wurden unter Benutzung des relativen Detriments für Frauen in der Altersklasse 18-65 Jahren berechnet, streng genommen entsprechen sie nicht der effektiven Dosis für die Referenzperson nach ICRP 103. Die hier angegeben Werte beziehen sich auf beidseitige Aufnahmen jeweils in einer Ebene; für einseitige Aufnahmen ist der Wert zu halbieren. Man beachte, dass das Organ Brust aus zwei Brustdrüsen besteht. Während der DRW pro Brustdrüse und Ebene gilt, geht in die Berechnung der effektiven Dosis die Organdosis der gesamten Brust ein.

Tabelle S3: DRW für konventionelle Projektionsaufnahmen am Kind

Untersuchungsart	Gewichts- / Altersklasse	*DFP* [a] [cGy·cm² = µGy·m²] *Medianwert*	*DRW*	Effektive Dosis [c] [mSv]
Thorax a.p./p.a. [b]	Frühgeborene (< 3 kg)	0,05	0,3	0,019
	Neugeborene (3 bis < 5 kg; 0 bis < 3 Monate)	0,11	0,5	0,021
	Säuglinge (5 bis < 10 kg; 3 bis <12 Monate)	0,19	0,8	0,014
	Frühe Kindheit (10 bis < 19 kg; 1 bis < 5 Jahre)	0,55	1,5	0,018
	Mittlere Kindheit (19 bis < 32 kg; 5 bis < 10 Jahre)	0,88	2,5	0,017
	Späte Kindheit (32 bis < 56 kg, 10 bis < 15 Jahre)	1,45	4,0	0,032
Abdomen a.p./p.a. [b]	Neugeborene (3 bis < 5 kg; 0 bis < 3 Monate)	0,04	1,5	0,041
	Säuglinge (5 bis < 10 kg; 3 bis <12 Monate)	0,04	5,0	0,064
	Frühe Kindheit (10 bis < 19 kg; 1 bis < 5 Jahre)	1,5	7,5	0,062
	Mittlere Kindheit (19 bis < 32 kg; 5 bis < 10 Jahre)	4,0	16	0,074
	Späte Kindheit (32 bis < 56 kg, 10 bis < 15 Jahre)	13	30	0,17
Becken a.p./p.a. [b]	Frühe Kindheit (10 bis < 19 kg; 1 bis < 5 Jahre)	1,7	12	0,088
	Mittlere Kindheit (19 bis < 32 kg; 5 bis < 10 Jahre)	4,8	25	0,093
	Späte Kindheit (32 bis < 56 kg, 10 bis < 15 Jahre)	9,5	50	0,24

[a] *DFP*: Dosis-Flächen-Produkt
[b] a.p.: anterior-posterior; p.a.: posterior-anterior.
[c] Die effektive Dosis bezieht sich auf a.p. Aufnahmen (konservative Abschätzung).

Tabelle S4: DRW für diagnostische Durchleuchtungsuntersuchungen am Erwachsenen

Untersuchungsart	*DFP* [a] [cGy·cm^2 = µGy·m^2]		Effektive Dosis [mSv]
	Medianwert	*DRW*	
Koronarangiographie	956	1800	4,9
Kolon Monokontrast	815	2000	4,1
Phlebographie Bein-Becken	196	400	0,3
Arteriographie Becken-Bein	1700	3500	4,3

[a] *DFP*: Dosis-Flächen-Produkt

Tabelle S5: DRW für diagnostische Durchleuchtungsuntersuchungen am Kind

Untersuchungsart	Gewichtsklasse bzw. Altersklasse	*DFP* [a] [cGy·cm² = µGy·m²]		Effektive Dosis [b]
		Medianwert	*DRW*	[mSv]
MCU [c]	Neugeborene (3 bis < 5 kg; < 3 Monate)	0,8	5	0,15
	Säuglinge (5 bis < 10 kg; 3 bis <12 Monate)	0,9	10	0,16
	Frühe Kindheit (10 bis < 19 kg; 1 bis < 5 Jahre)	0,9	18	0,16
	Mittlere Kindheit (19 bis < 32 kg; 5 bis < 10 Jahre)	1,8	30	0,17

[a] *DFP*: Dosis-Flächen-Produkt

[b] Diese Werte sind mit wesentlichen Unsicherheiten im Zusammenhang mit der Festlegung der Expositionsbedingungen und mit der Berechnung der Konversionsfaktoren behaftet.

[c] MCU: Miktions-Cysto-Urographie

Tabelle S6: DRW für interventionelle Eingriffe am Erwachsenen [a]

Art des interventionellen Eingriffs	*DFP* [b] [cGy·cm² = µGy·m²]		Effektive Dosis [mSv]
	Medianwert	*DRW*	
Endovaskuläre Behandlung des akuten Schlaganfalls (Thrombektomie)	6940	14000	8,7
Endovaskuläre Behandlung eines Hirnarterienaneurysmas	8750	20000	9,6
PCI [c]	2250	3500	9,8
Kombinierte Koronarangiographie/PCI	2460	4000	11
TAVI [d]	3300	5000	14
EVAR [e]			
einfache Prothesen	5600	20000	46
fenestrierte/gebranchte Prothesen	16500	30000	68
TACE [f]	9400	20000	52
PTA [g] Becken	2420	5000	12
PTA Oberschenkel-Knie	1070	2500	1,2
PTA Unterschenkel-Fuß	795	1800	0,1
ERCP [h] (Intervention)	620	2000	5,4

[a] Bei interventionellen Strahlenanwendungen ist ein gescheiterter Eingriff (frustrane Implantation) als Therapie zu berücksichtigen
[b] *DFP*: Dosis-Flächen-Produkt
[c] Perkutane koronare Intervention
[d] Transkatheter Aortenklappen-Implantation
[e] Endovaskuläre Aneurysma-Therapie
[f] Transarterielle Chemoembolisation
[g] Perkutane transluminare Angioplastie
[h] Endoskopisch retrograde Cholangiopankreatikographie

Tabelle S7: DRW für elektrophysiologische Untersuchung (EPU) am Erwachsenen [a]

Art der Implantation	*DFP* [b] [cGy·cm^2 = µGy·m^2]	Effektive Dosis [mSv]
Einkammersystem	900	2,5
Zweikammersystem	1700	4,8
Dreikammersystem	4900	13,7

[a] Die Werte gelten sowohl für die Implantation von Schrittmachern als auch von Defibrillatoren. Die Werte sind auf Basis von Expertenmeinungen festgelegt worden, Medianwerte nicht vorhanden.

[b] *DFP*: Dosis-Flächen-Produkt

Tabelle S8: DRW für CT-Untersuchungen am Erwachsenen

Untersuchungsregion	*CTDI*vol [a] [mGy] *Medianwert*	*DRW*	Effektive Dosis [mSv]
Gehirn	48	55	1,4
Gesichtsschädel (Tumordiagnostik, Trauma)	13	20	0,9
NNH[b] (Sinusitis, OP-Planung)	5,0	7	0,2
Hals	9,9	15	2,9
CT-Angiographie der hirnversorgenden Gefäße	8,5	15	4,5
Halswirbelsäule (Bandscheibe)	15	23	2,6
Halswirbelsäule (Knochen)	12	15	1,7
Brustwirbelsäule (Bandscheibe)	16	23	18
Brustwirbelsäule (Knochen)	9,7	15	12
Lendenwirbelsäule (Bandscheibe)	18	23	8,9
Lendenwirbelsäule (Knochen)	10	15	5,8
Thorax Weichteile	6,2	8	6,1
Lungenparenchym	2,4	3	2,3
CT-Angiographie der gesamten Aorta	7,3	10	12
EKG-synchronisierte koronare Angiographie [c]	13	20	7,6
Abdomen (z. B. Leber, Pankreas)	8,0	12	7,0
Abdomen mit Becken	9,0	12	9,7
Rumpf (Thorax+Abdomen+Becken)	8,0	12	16
Becken (Weichteile)	9,9	12	3,5
Becken (Knochen)	8,4	10	2,1
CT-Angiographie Becken-Bein	5,0	7	8,3

[a] Die angegebenen *CTDI*vol-Werte für Untersuchungen am Hirn- und Gesichtsschädel sowie der Nasennebenhöhlen beziehen sich auf den 16 cm-CTDI-Prüfkörper („Kopfphantom“); alle anderen Angaben auf den 32 cm-CTDI-Prüfkörper („Körperphantom“).

[b] NNH: Nasennebenhöhlen

[c] Die retrospektive Variante sollte nur unter besonderen Umständen durchgeführt werden (z. B. Patienten mit Arrhythmie). Die daraus ggf. resultierende Überschreitung der DRW ist zu begründen.

Tabelle S9: DRW für CT-Untersuchungen am Kind

Untersuchungsregion	Gewichts- bzw. Altersklasse	$CTDI_{vol}$ [b] [mGy] *Medianwert*	*DRW*	Effektive Dosis [mSv]
Gehirn (Schädelbasis – Kalotte) [a]	Säuglinge (3 bis < 12 Monate)	10	25	2,1
	Frühe Kindheit (1 bis < 5 Jahre)	17	35	2,8
	Mittlere Kindheit (5 bis < 10 Jahre)	17	40	2,0
	Späte Kindheit (10 bis < 15 Jahre)	29	45	1,8
Thorax (Lungenspitze - dorsaler Recessus)	Neugeborene (3 bis < 5 kg; 0 bis < 3 Monate)	0,3	1,0	0,8
	Säuglinge (5 bis < 10 kg; 3 bis <12 Monate)	0,5	1,5	1,3
	Frühe Kindheit (10 bis < 19 kg; 1 bis < 5 Jahre)	0,6	2,0	1,5
	Mittlere Kindheit (19 bis < 32 kg; 5 bis < 10 Jahre)	0,8	3,0	1,8
	Späte Kindheit (32 bis < 56 kg; 10 bis < 15 Jahre)	1,4	5,0	2,9
Abdomen (Obere Zwerchfellkuppe -Symphyse)	Mittlere Kindheit (19 bis < 32 kg; 5 bis < 10 Jahre)	1,9	4,0	4,2
	Späte Kindheit (32 bis < 56 kg; 10 bis < 15 Jahre)	5,0	7,0	6,1

[a] Bei Kindern / Jugendlichen können je nach Wachstumsschub die Scanlängen erheblich variieren.
[b] Die angegebenen $CTDI_{vol}$-Werte für Untersuchungen am Hirnschädel beziehen sich auf den 16 cm-CTDI-Prüfkörper („Kopfphantom“). Die anderen Untersuchungen auf den 32 cm-CTDI-Prüfkörper („Körperphantom“).

Tabelle S10: DRW für DVT [a]-Untersuchungen am Erwachsenen

Untersuchungsart	*DFP* [b] [mGy·cm²] *Medianwert*	*DRW*	Effektive Dosis [mSv]
Nasennebenhöhlen (NNH)	1100	1500	0,22
Dental [c]			
FOV [d] ≤ 5 [e] x 5 [f] (≤ 25 cm²)	330	500	0,12
FOV [d] > 5 [e] x 5 [f] (> 25 cm²)	600	1000	0,17

[a] Digitale Volumentomographie (auch Cone-Beam-CT genannt)

[b] *DFP*: Dosis-Flächen-Produkt. Man beachte, dass üblicherweise bei DVT-Untersuchungen mGy cm² die international verwendete Einheit für die Angabe des DFP ist.

[c] Werden auch endodontische Untersuchungen in einer Einrichtung durchgeführt, so kann es zu einer beständigen aber gerechtfertigten Überschreitung des DRW kommen. Dies ist zu dokumentieren.

[d] FOV: Field-of-View

[e] Transaxiale Einblendung

[f] Axiale Einblendung
