# Supplementary material for: Reference Levels for Diagnostic and Interventional X-ray Exposures in Germany: Development, Handling, and Perspective
Source: Rofo. 2026 Mar 16;198(11):1444–51. doi: 10.1055/a-2790-4742 (PMC13716514; doi:10.1055/a-2790-4742)
Supplement: Supplementary file 2 [file 10-1055-a-2790-4742_28411431.pdf]

Supplement

Compilation of the diagnostic reference levels (DRLs) published in 2022, along with the corresponding effective dose values, and for guidance, the median values of the exposure levels collected nationwide from operators.

Table S1: DRLs for conventional projection radiography on adults

Type of examination	*DAP* [a] [cGy·cm² = µGy·m²]		Effective dose [mSv]
	Median value	*DRLs*	
Shoulder (per plane) [b]	13	25	0.009
Thorax p.a.[c]	8	12	0.030
Thorax a.p.[c] (supine)	9	15	0.075
Thorax lat.[c]	22	40	0.11
Thoracic spine a.p./p.a.[d]	60	100	0.43
Thoracic spine lat.	65	120	0.17
Lumbar spine a.p./p.a.[d]	122	200	0.54
Lumbar spine lat.	200	330	0.34
Abdomen a.p./p.a.[d]	123	200	0.51
Pelvis a.p./p.a.[d]	145	230	0.36
Hips (per plane)	57	100	0.093

[a] *DAP*: Dose Area Product

[b] In studies performed in two planes, the dose area product (DAP) is 50 cGy cm², even if a Y-view is included.

[c] a. p.: anterior-posterior; p. a.: posterior-anterior; lat.: lateral

[d] According to the German Medical Association's guidelines (https://doi.org/10.3238/arztebl.2023.LL_Qualitaetssicherung_Roentgendiagnostik), only a.p. views are recommended.

Table S2: DRLs for mammography examinations

Type of examination	*AGD* [a] [mGy]		Effective dose[b] [mSv]
	Median value	*DRLs*	
Mammography (per plane)	1.4	2.0	0.28
Tomosynthesis (per plane)	1.9	2.5	0.34

[a] AGD: average glandular dose (average parenchymal dose)

[b] The values were calculated using the relative detriment for women in the age group 18-65 years; strictly speaking, they do not correspond to the effective dose for the reference person according to ICRP 103. The values given here refer to bilateral exposures in a single plane; for unilateral exposures, the value should be halved. Note that the breast organ consists of two mammary glands. While the DRLs apply to each mammary gland and plane, the organ dose for the entire breast is used to calculate the effective dose.

Table S3: DRLs for conventional projection radiography on children

Type of examination	Weight/age group	*DAP* [a] [cGy·cm² = µGy·m²] *Median value*	*DRLs*	Effective dose[c] [mSv]
Thorax a.p./p.a.[b]	Premature babies (< 3 kg)	0.05	0.3	0.019
	Newborns (3 to < 5 kg; 0 to < 3 months)	0.11	0.5	0.021
	Infants (5 to < 10 kg; 3 to < 12 months)	0.19	0.8	0.014
	Early childhood (10 to < 19 kg; 1 to < 5 years)	0.55	1.5	0.018
	Middle childhood (19 to < 32 kg; 5 to < 10 years)	0.88	2.5	0.017
	Late childhood (32 to < 56 kg, 10 to < 15 years)	1.45	4.0	0.032
Abdomen a.p./p.a.[b]	Newborns (3 to < 5 kg; 0 to < 3 months)	0.04	1.5	0.041
	Infants (5 to < 10 kg; 3 to < 12 months)	0.04	5.0	0.064
	Early childhood (10 to < 19 kg; 1 to < 5 years)	1.5	7.5	0.062
	Middle childhood (19 to < 32 kg; 5 to < 10 years)	4.0	16	0.074
	Late childhood (32 to < 56 kg, 10 to < 15 years)	13	30	0.17
Pelvis a.p./p.a.[b]	Early childhood (10 to < 19 kg; 1 to < 5 years)	1.7	12	0.088
	Middle childhood (19 to < 32 kg; 5 to < 10 years)	4.8	25	0.093
	Late childhood (32 to < 56 kg, 10 to < 15 years)	9.5	50	0.24

[a] *DAP*: Dose Area Product

[b] a.p.: anterior-posterior; p.a.: posterior-anterior.

[c] The effective dose refers to a.p. imaging (conservative estimate).

Table S4: DRLs for diagnostic fluoroscopic examinations on adults

Type of examination	*DAP* [a] [cGy·cm^2 = µGy·m^2]		Effective dose [mSv]
	Median value	*DRLs*	
Coronary angiography	956	1,800	4.9
Colon mono-contrast	815	2,000	4.1
Phlebography of the leg-pelvis	196	400	0.3
Arteriography of the pelvis-leg	1,700	3,500	4.3

[a] *DAP*: Dose Area Product

Table S5: DRLs for diagnostic fluoroscopic examinations on children

Type of examination	Weight group or age group	*DAP* [a] [cGy·cm^2 = µGy·m^2]		Effective dose[b] [mSv]
		Median value	*DRLs*	
MCU[c]	Newborns (3 to < 5 kg; < 3 months)	0.8	5	0.15
	Infants (5 to < 10 kg; 3 to < 12 months)	0.9	10	0.16
	Early childhood (10 to < 19 kg; 1 to < 5 years)	0.9	18	0.16
	Middle childhood (19 to < 32 kg; 5 to < 10 years)	1.8	30	0.17

[a] *DAP*: Dose Area Product
[b] These values are subject to significant uncertainties related to the determination of the exposure conditions and the calculation of the conversion factors.
[c] MCU: micturating cystourethrography

Table S6: DRLs for interventional procedures on adults[a]

Type of interventional procedure	*DAP*[b] [cGy·cm² = µGy·m²] *Median value*	*DRLs*	Effective dose [mSv]
Endovascular treatment of acute stroke (thrombectomy)	6,940	14,000	8.7
Endovascular treatment of a cerebral artery aneurysm	8,750	20,000	9.6
PCI[c]	2,250	3,500	9.8
Combined coronary angiography/PCI	2,460	4,000	11
TAVI[d]	3,300	5,000	14
EVAR[e]			
Simple prostheses	5,600	20,000	46
Fenestrated/branched prostheses	16,500	30,000	68
TACE[f]	9,400	20,000	52
PTA[g] pelvis	2420	5,000	12
PTA thigh and knee	1070	2,500	1.2
PTA lower-leg and foot	795	1,800	0.1
ERCP[h] (intervention)	620	2,000	5.4

[a] In interventional radiation procedures, a failed intervention (unsuccessful implantation) should be considered as therapy
[b] *DAP*: Dose Area Product
[c] Percutaneous coronary intervention
[d] Transcatheter aortic valve implantation
[e] Endovascular aneurysm therapy
[f] Transarterial chemoembolization
[g] Percutaneous transluminal angioplasty
[h] Endoscopic retrograde cholangiopancreatography

Table S7: DRLs for electrophysiology study (EPS) on adults[a]

Type of implantation	*DAP* [b] [cGy·cm² = µGy·m²]	Effective dose [mSv]
Unicameral system	900	2.5
Bicameral system	1,700	4.8
Tricameral system	4,900	13.7

[a] The values apply to the implantation of both pacemakers and defibrillators. The values have been determined based on expert opinions; median values are not available.

[b] *DAP*: Dose Area Product

Table S8: DRLs for CT studies on adults

Examination area	*CTDI*vol [a] [mGy] *Median value*	*CTDI*vol [a] [mGy] *DRLs*	Effective dose [mSv]
Brain	48	55	1.4
Facial skull (tumor diagnostics, trauma)	13	20	0.9
PNS[b] (sinusitis, surgery planning)	5.0	7	0.2
Neck	9.9	15	2.9
CT angiography of the blood vessels supplying the brain	8.5	15	4.5
Cervical spine (intervertebral disc)	15	23	2.6
Cervical spine (bone)	12	15	1.7
Thoracic spine (intervertebral disc)	16	23	18
Thoracic spine (bone)	9.7	15	12
Lumbar spine (intervertebral disc)	18	23	8.9
Lumbar spine (bone)	10	15	5.8
Thorax soft tissues	6.2	8	6.1
Lung parenchyma	2.4	3	2.3
CT angiography of the entire aorta	7.3	10	12
ECG-synchronized coronary angiography[c]	13	20	7.6
Abdomen (e.g. Liver, pancreas)	8.0	12	7.0
Abdomen with pelvis	9.0	12	9.7
Trunk (thorax + abdomen + pelvis)	8.0	12	16
Pelvis (soft tissues)	9.9	12	3.5
Pelvis (bone)	8.4	10	2.1
CT angiography of the pelvis and leg	5.0	7	8.3

[a] The *CTDI*vol values provided for examinations of the brain, facial skull, and paranasal sinuses refer to the 16-cm CTDI dosimetry phantom (“head phantom”); all other values refer to the 32-cm CTDI dosimetry phantom (“body phantom”).

[b] PNS: paranasal sinuses

[c] The retrospective method should only be used under special circumstances (e.g. patients with arrhythmia). Any resulting values that exceed the DRLs must be justified.

Table S9: DRLs for CT studies on children

Examination area	Weight or age group	$CTDI_{vol}$ [b] [mGy] Median value	DRLs	Effective dose [mSv]
Brain (base of skull – calvarium)[a]	Infants (3 to < 12 months)	10	25	2.1
	Early childhood (1 to < 5 years)	17	35	2.8
	Middle childhood (5 to < 10 years)	17	40	2.0
	Late childhood (10 to < 15 years)	29	45	1.8
Thorax (tip of the lung - dorsal recess)	Newborns (3 to < 5 kg; 0 to < 3 months)	0.3	1.0	0.8
	Infants (5 to < 10 kg; 3 to < 12 months)	0.5	1.5	1.3
	Early childhood (10 to < 19 kg; 1 to < 5 years)	0.6	2.0	1.5
	Middle childhood (19 to < 32 kg; 5 to < 10 years)	0.8	3.0	1.8
	Late childhood (32 to < 56 kg; 10 to < 15 years)	1.4	5.0	2.9
Abdomen (upper diaphragm dome - symphysis)	Middle childhood (19 to < 32 kg; 5 to < 10 years)	1.9	4.0	4.2
	Late childhood (32 to < 56 kg; 10 to < 15 years)	5.0	7.0	6.1

[a] In children and adolescents, the scan lengths can vary considerably depending on growth spurts.
[b] The $CTDI_{vol}$ values provided for examinations of the cranium refer to the 16-cm CTDI dosimetry phantoms ("head phantom"). The other examinations were performed on the 32-cm CTDI dosimetry phantoms ("body phantom").

Table S10: DRLs for CBCT[a] studies on adults

Type of examination	*DAP* [b] [mGy·cm²] *Median value*	*DRLs*	Effective dose [mSv]
Paranasal sinuses (PNS)	1,100	1,500	0.22
Dental[c]			
FOV[d] ≤ 5[e] x 5[f] (≤ 25 cm²)	330	500	0.12
FOV[d] > 5[e] x 5[f] (> 25 cm²)	600	1,000	0.17

[a] Digital volume tomography (i.e. cone beam CT (CBCT))

[b] *DAP*: Dose Area Product. Please note that mGy cm² is the internationally accepted unit for specifying the DAP in CBCT examinations.

[c] If endodontic examinations are also performed at a facility, the value may exceed the diagnostic reference level (DRL) on a persistent but justified basis. This must be documented.

[d] FOV: Field-of-View

[e] Transaxial field-of-view

[f] Axial field-of-view
