# Supplementary material for: Reference Levels for Diagnostic and Interventional X-ray Exposures in Germany: Development, Handling, and Perspective
Source: Rofo. 2026 Mar 16;198(11):1444–51. doi: 10.1055/a-2790-4742 (PMC13716514; doi:10.1055/a-2790-4742)
Supplement: Related texts

# Research article: Referenzwerte für diagnostische und interventionelle Röntgenanwendungen in Deutschland: Entwicklung, Handhabung und Perspektive

## Abstract

**Ziel:**

Diagnostische Referenzwerte (DRW) dienen der Optimierung von diagnostischen und interventionellen Strahlenexpositionen, die in Deutschland mit über 95% zur zivilisatorischen Strahlenexposition der Bevölkerung beitragen. Die national gültigen DRW werden regelmäßig an die Entwicklung der Gerätetechnologie und der klinischen Praxis angepasst. Ziel des vorliegenden Beitrags ist es, das grundlegende Konzept der DRW, seine methodische Fortentwicklung und der dadurch erzielten Verbesserung des Patientenschutzes darzustellen.

**Materialien und Methoden:**

Die einschlägigen Regelungen des neuen Strahlenschutzrechts in Deutschland werden zusammengefasst, die Entwicklung der DRW zwischen 2003 und 2022 wird analysiert, Hinweise für die praktische Anwendung der DRW und die Umsetzung ergänzender einrichtungsspezifischer Maßnahmen sowie ein Ausblick auf zukünftige Fragestellungen werden gegeben.

**Ergebnisse:**

Seit der ersten amtlichen Bekanntmachung von DRW in Deutschland konnten diese durch kontinuierliche Protokolloptimierungen und den Einsatz modernerer Hard- und Software erheblich reduziert werden. Über den betrachteten Zeitraum von fast 20 Jahren wurde der Katalog mit Röntgenanwendungen, für die DRW festgelegt wurden, erheblich erweitert, obwohl für verschiedene nicht mehr regelhaft indizierte Anwendungen DRW entfernt wurden.

**Schlussfolgerung:**

Die konsequente Umsetzung des Konzepts der DRW hat sich in der Praxis bewährt und über die Jahre zu einer erheblichen Reduktion der Patientenexposition geführt. Diese positive Entwicklung im Sinne des Patientenschutzes rechtfertigt den personellen Aufwand sowie die ggf. erforderliche Erneuerung von Hard- und Software. Trotz der erzielten Erfolge bedarf das DRW-Konzept auch zukünftig der kontinuierlichen Fortentwicklung.

**Kernaussagen:**

- Diagnostische Referenzwerte (DRW) sind ein effektives Instrument zur Optimierung von Röntgenanwendungen.
- In Deutschland konnten viele DRW in annähernd 20 Jahren mehr als halbiert werden.
- DRW sollen und können bei guter Ausstattung und Praxis deutlich unterschritten werden.
- Dosismanagementsysteme erleichtern die praktische Umsetzung des DRW-Konzepts erheblich.
- Die national festgelegten DRW sollten durch einrichtungsspezifisch festgelegte lokale Werte ergänzt werden.

**Zitierweise:**

- de las Heras Gala H, Giussani A, Schlattl H et al. Reference Levels for Diagnostic and Interventional X-ray Exposures in Germany: Development, Handling, and Perspective. Rofo 2026; DOI 10.1055/a-2790-4742

## Einleitung

In Deutschland tragen diagnostische und interventionelle Röntgenanwendungen mit mehr als 95% zur zivilisatorischen Strahlenexposition der Bevölkerung bei. Daher kommt der konsequenten Anwendung der beiden Grundprinzipien des medizinischen Strahlenschutzes, nämlich der Rechtfertigung und Optimierung, ein hoher Stellenwert zu. Das gilt insbesondere für dosisintensivere Anwendungen, vor allem für CT-Untersuchungen sowie Angiografien und vaskuläre Interventionen, die 2021 mit etwa 71% bzw. 14% zur medizinischen Strahlenexposition der Bevölkerung durch Röntgenanwendungen beitrugen – obwohl sie nur etwa 11% bzw. 3% der Gesamthäufigkeit medizinischer Röntgenanwendungen ausmachten 1 2 .

Als zentrales Element der Optimierung wurde von der International Commission on Radiological Protection (ICRP) das Konzept der Diagnostischen Referenzwerte (DRW) vorgeschlagen und nachfolgend in der Europäischen Union sowie in Deutschland rechtlich verankert. DRW werden für häufige und/oder dosisintensivere Röntgenanwendungen festgelegt. Sie sollen bei üblicher Geräteausstattung bezogen auf eine Gruppe von Patienten eingehalten werden, stellen jedoch keine Grenzwerte dar. Bei moderner Gerätausstattung können und sollten sie deutlich unterschritten werden. Werden die jeweils aktuellen DRW nicht eingehalten, so ist eine sorgfältige Optimierung der verwendeten Protokolle und im Extremfall eine Modernisierung der Geräteausstattung erforderlich. Die DRW sind in regelmäßigen Zeitabständen basierend auf repräsentativen Datenerhebungen zu aktualisieren, um u.a. der rasanten Entwicklung der Gerätetechnologie Rechnung zu tragen. Bei der regelmäßigen Aktualisierung ist auch zu prüfen, ob aufgrund neuer Gerätetechnologien und/oder der Etablierung neuer Untersuchungsarten DRW für nicht mehr regelhaft indizierte Anwendungen entfallen können bzw. neue festzulegen sind. Eine Zusammenstellung der derzeit gültigen DRW sowie – informationshalber – der korrespondierenden Werte der effektiven Dosis ist in den **Tabellen S1-S10 als Zusatzmaterial (Supplement)** beigefügt.

Die vorliegende Übersichtsarbeit

- thematisiert Veränderungen des Konzepts der DRW und der entsprechenden Anpassungen im deutschen Strahlenschutzrecht;
- verdeutlicht die erzielten Erfolge hinsichtlich der angestrebten Reduktion der Patientenexposition im Laufe der Jahre;
- gibt Hinweise zur konkreten Anwendung einiger aktueller DRW;
- macht Vorschläge für einrichtungsspezifische Maßnahmen zur Qualitätssicherung von Röntgenanwendungen, die die bundeseinheitliche Umsetzung des Konzepts der nationalen DRW unterstützen und ergänzen.

## ICRP-Empfehlungen zum Konzept der DRW und dessen rechtliche und praktische Umsetzung in Deutschland

Das Konzept der DRW wurde im Jahre 1996 erstmals von der ICRP 3 eingeführt und in mehreren Nachfolgeveröffentlichungen konkretisiert und fortentwickelt 4 5 6 . So wurde in der letzten Veröffentlichung empfohlen, zur Charakterisierung der stichprobenartig bei Betreibern zu erhebenden Dosisverteilungen Medianwerte zu verwenden (s.u.), da dafür keine Annahmen über die Verteilung der Stichprobenwerte vorausgesetzt werden, während das in der Praxis bislang häufiger verwendete arithmetische Mittel nur im Falle einer Normalverteilung einen sinnvollen Schätzer des Erwartungswerts liefert. Darüber hinaus wird der Medianwert weniger von Ausreißern beeinflusst.

Das Konzept der DRW wurde auf europäischer Ebene bereits 1997 mit der Richtlinie 97/43/EURATOM eingeführt 7 und in der Richtlinie 2013/59/EURATOM präzisiert und konsolidiert 8 . In Deutschland wurde das Konzept bereits in der Strahlenschutzverordnung des Jahres 2001 (§81, Nummer 2) 9 und der Röntgenverordnung des Jahres 2003 (§16, Nummer 1) 10 verbindlich eingeführt sowie mit dem am 31.12.2018 in Kraft getretenen neuen Strahlenschutzgesetz (StrlSchG) 11 und der zugehörigen neuen Strahlenschutzverordnung (StrlSchV) 12 fortgeführt. Terminologisch ist zu beachten, dass in der deutschen Rechtsetzung diagnostische und interventionelle Röntgenanwendungen unter dem Oberbegriff „Untersuchungen“ zusammengefasst werden.

Wie in Abb. 1 verdeutlicht, sind in Deutschland im Wesentlichen drei Akteure an der Umsetzung des Konzepts beteiligt: das Bundesamt für Strahlenschutz (BfS), die Betreiber medizinischer Röntgengeräte sowie die Ärztlichen oder Zahnärztlichen Stellen (ÄS bzw. ZahnÄS).

**Abb. 1:** Schematische Darstellung des in Deutschland realisierten zyklischen Konzepts der DRW. Die zentralen Aufgaben der involvierten drei Akteure sind kurz benannt; weitergehende Details werden im Text erläutert.

Nach § 122 Absatz 3 StrlSchV sind seitens der Betreiber (vertreten durch den jeweiligen Strahlenschutzverantwortlichen, d.h. die für den Strahlenschutz gesamtverantwortlich handelnde Person) die vom BfS publizierten DRW bei Untersuchungen von Personen zugrunde zu legen. Wie bereits erwähnt, stellen sie keine Grenzwerte für die Untersuchung von Einzelpersonen dar, sondern beziehen sich auf eine Gruppe von Patient*innen, die sich der jeweiligen Untersuchung unterziehen.

Durch die zuständigen ÄS bzw. ZahnÄS ist die Einhaltung der DRW zu prüfen. Hierzu sind im Rahmen ihrer routinemäßigen Überprüfungen für verschiedene Untersuchungsarten von den Betreibern jeweils für mindestens 10 Untersuchungen 1 die entsprechenden operationellen Expositionsparameter anzufordern, hieraus die zugehörigen Medianwerte zu berechnen und diese mit dem jeweiligen DRW zu vergleichen, wie im „Leitfaden zur Handhabung der diagnostischen Referenzwerte für diagnostische und interventionelle Röntgenanwendungen“ beschrieben 13 .

Es ist anzumerken, dass die Stichprobenerhebung der ÄS bei den Betreibern nicht einheitlich erfolgt. So fordern einige Expositionsdaten für Röntgenanwendungen an, die den Betreibern entweder anhand des Röntgentagebuches vorgegeben oder die in einem vorgegebenen Zeitraum durchgeführt wurden. Andere verlangen Daten für eine bestimmte Anzahl konsekutiv durchgeführter Röntgenanwendungen. Es gibt aber auch ÄS, die ganz auf konkrete Vorgaben verzichten und den Betreibern die Auswahl der zu übermittelnden Expositionsdaten überlassen. Letzteres birgt allerdings die Gefahr, dass gezielt niedrige Expositionswerte gemeldet werden.

Im Fall einer beständigen, ungerechtfertigten Überschreitung der DRW meldet die ÄS bzw. ZahnÄS dies der zuständigen Behörde (§ 130 Absatz 3 Satz 1 Nummer 3 StrlSchV). Es ist zu betonen, dass die Einhaltung der DRW nicht das Optimierungsgebot nach § 83 Absatz 5 StrlSchG ersetzt. Die DRW sind keine Optimal- oder Zielwerte, sondern können und sollen unterschritten werden, wenn die erreichte Bildqualität für die diagnostische Bewertung ausreicht. Falls bei Betreibern Optimierungspotenzial besteht, so beraten die ÄS bzw. ZahnÄS sie entsprechend.

Die DRW sind regelmäßig an den Stand der Technik und der medizinischen Wissenschaft anzupassen. Hierzu sind nach § 125 Absatz 1 Satz 3 StrlSchV die von den ÄS und ZahnÄS erhobenen Werte der Patientenexpositionen dem BfS jährlich in anonymisierter Form zu übermitteln. Dieses prüft laut § 125 Absatz 2 StrlSchV spätestens alle 3 Jahre, ob die DRW angepasst werden müssen und aktualisiert sie gegebenenfalls. Die Festlegung aktualisierter DRW orientiert sich jeweils am 3. Quartil der Medianwerte der von den ÄS bzw. ZahnÄS übermittelten Daten. Für die initiale Festlegung neuer DRW, für die konzeptbedingt noch keine Daten der ÄS oder ZahnÄS zur Verfügung stehen, werden operationelle Dosiswerte aus anderen nationalen Quellen und Erhebungen herangezogen 14 .

Aufgrund der beschriebenen Methodik der Datenverarbeitung beziehen sich die statistischen Kenngrößen – entgegen einer des Öfteren geäußerten Meinung – nicht auf die relativ schlanken Referenzpersonen der ICRP, sondern auf typische Patient*innen. Hiervon ausgehend findet dann ein umfassendes Konsultationsverfahren mit Vertretern relevanter Stakeholder statt, um sicher zu stellen, dass alle für die Anwendung relevanten klinischen Aspekte berücksichtigt werden. Unter Abwägung aller diskutierten Aspekte legt das BfS dann abschließend die DRW fest und macht diese im Bundesanzeiger bekannt.

## Zeitliche Entwicklung der DRW in Deutschland

Nationale DRW für Röntgenanwendungen wurden in Deutschland erstmals 2003 15 veröffentlicht und 2010 16 , 2016 17 , 2018 18 und 2022 19 an den jeweiligen Stand der radiologischen Technik und klinischen Praxis angepasst. So wurden einerseits eine ganze Reihe von neuen DRW festgelegt (u.a. für die dentale und maxillofaziale Digitale Volumentomografie, DVT, sowie die Tomosynthese der Brust) und andererseits DRW für Untersuchungen aus dem Katalog entfernt, die nicht mehr dem aktuellen Stand der Praxis entsprachen und nur noch in Sonderfällen indiziert sind (u.a. pädiatrische Röntgenaufnahmen des Schädels). Aktuell gibt es für diagnostische und interventionelle Röntgenanwendungen insgesamt 52 DRW für Erwachsene (2003: 25) und 7 für Kinder und Jugendliche (2003: 7), wobei letztere in bis zu 6 Gewichts-/Altersklassen unterteilt sind.

Die zeitliche Entwicklung der DRW lässt sich gut anhand der Untersuchungen verfolgen, für die bei Erwachsenen die DRW nach der Erstveröffentlichung 2003 mindestens dreimal ( Abb. 2 ) und für Kinder/Jugendliche nach der Erstveröffentlichung 2003 oder 2010 mindestens zweimal ( Abb. 3 ) aktualisiert wurden. Zum Vergleich sind – soweit vorhanden und vergleichbar – die entsprechenden europäischen DRW 20 21 22 dargestellt.

**Abb. 2:** Zeitliche Entwicklung der DRW in Deutschland (gefüllte farbige Symbole) für vergleichbare Röntgenanwendungen an Erwachsenen, für die zwischen 2003 und 2022 bereits vier DRW festgelegt wurden. **a** Planare Röntgenaufnahmen, **b** Durchleuchtungen und Interventionen und **c** Computertomografien. Zum Vergleich sind – soweit vorhanden und vergleichbar – die entsprechenden europäischen DRW 20 21 dargestellt (offene farbige Symbole). Hinweis: Die in der ersten Bekanntmachung der deutschen DRW (2003) für die CT festgelegten CTDI _vol_ -Werte bezogen sich nicht wie in den nachfolgenden Bekanntmachungen auf Prä- oder Postkontrastscans, sondern berücksichtigten, dass bei einem Teil der Untersuchungen beide Scans akquiriert werden. Um einen fairen Vergleich zu ermöglichen, wurden diese Werte daher auf einen einzelnen Scan umgerechnet.

**Abb. 3:** Zeitliche Entwicklung der DRW in Deutschland (gefüllte farbige Symbole) für vergleichbare Röntgenanwendungen bei Kindern, für die zwischen 2003 bzw. 2010 und 2022 mindestens drei DRW festgelegt wurden. Die Daten beziehen sich exemplarisch auf die Gewichtsgruppe „Mittlere Kindheit“ (19 bis <32 kg, ca. 5 bis 10 Jahre); **a** Planare Röntgenaufnahmen, **b** Miktionszystourethrografie (MCU) und **c** Computertomografie. Zum Vergleich sind entsprechende europäische DRW aus dem Jahr 2018 22 dargestellt (offene farbige Symbole).

Wie die vorgenannten Abbildungen veranschaulichen, ist für die allermeisten Anwendungen über die Jahre hinweg eine erhebliche Reduktion der DRW zu verzeichnen, die meist anfänglich recht deutlich war und dann etwas abflachte. Die anfänglich besonders ausgeprägte Reduktion ist auf zwei Effekte zurückzuführen: (i) Einer noch nicht ausreichenden und repräsentativen Datenbasis bei der erstmaligen Festlegung der DRW, sodass diese teilweise nicht zu restriktiv angesetzt wurden. (ii) Die Einführung der DRW hat bei vielen Betreibern dazu geführt, das initial bestehende erhebliche Dosisreduktionspotenzial durch konsequente Protokolloptimierungen zu realisieren, wohingegen nachfolgende Reduktionen vor allem durch die kontinuierliche Erneuerung der Hard- und Software im Markt getrieben wurde. Aufgrund der rasanten technologischen Fortschritte im Bereich der medizinischen Bildgebung ist zu erwarten, dass sich der letztgenannte Trend fortsetzen wird.

## Konzeptionelle Änderungen der letzten Aktualisierung (2022)

Basierend auf den gesammelten Erfahrungen hat die Festlegung von DRW auch in Deutschland über die Jahre hinweg verschiedene Modifikationen erfahren. Die wesentlichen Veränderungen bei der letzten Aktualisierung betrafen folgende Aspekte:

- Für CT-Untersuchungen wurden DRW nur noch auf Basis des Volumen-CT-Dosisindex ( *CTDI*_vol_ ) festgelegt und nicht mehr für das Dosislängenprodukt ( *DLP* ) als primärer Größe. Hintergrund war, dass das *DLP* aufgrund der berücksichtigten Scanlänge wesentlich stärker als der *CTDI*_vol_ von der klinischen Fragestellung und der daraus resultierenden rechtfertigenden Indikation abhängt. Auch deshalb konnte beispielsweise für CT-Untersuchungen der Wirbelsäule (HWS, BWS und LWS) ein einheitlicher DRW festgelegt werden. Zur Orientierung wurden aber typische Scanlängen für die jeweiligen Untersuchungen in der Bekanntmachung mit veröffentlicht. Es wird allerdings empfohlen, einrichtungsspezifische Alarmschwellen auf Basis des *DLP* beizubehalten.
- Prospektiv getriggerten und retrospektiv EKG-synchronisierten Koronarangiografien wurde ein einheitlicher DRW zugeordnet, wobei retrospektiv synchronisierte Untersuchungen nur noch unter besonderen Umständen durchgeführt werden sollten (z.B. bei Patienten mit Arrhythmien).
- DRW für die Aneurysmatherapie (EVAR) wurden unter Berücksichtigung ihrer Komplexität festgelegt, und zwar jeweils ein Wert für einfache und komplexe/gebranchte Prothesen.
- Für die Implantation von Herzschrittmachern oder Defibrillatoren wurden ebenfalls separate DRW entsprechend der Komplexität der Eingriffe, d.h. anhand der Anzahl der involvierten Kammern, festgelegt.

## Hinweise zur Anwendung der DRW

Im Folgenden werden konkretisierende Erläuterungen zur Anwendung verschiedener DRW gegeben.

- *CT-Untersuchungen mehreren Körperregionen* : Bei der Untersuchung mehrerer Körperregionen sollten, wenn irgend möglich, separate Scans durchgeführt werden, die jeweils die für die jeweilige Körperregion/Indikation festgelegten DRW beachten (konform mit der Leitlinie der Bundesärztekammer 23 ). Wenn die Kombination von mehreren Körperteilen in einem Scan klinisch indiziert ist (z.B. bei Unfallpatienten), so ist der *CTDI*_vol_ für den Körperteil mit dem höchsten DRW einzuhalten.
- *DRW für dentale DVT:* Für den Fall von besonders großen Volumina, die nicht nur die Mundhöhle abdecken, steht aktuell eine zu geringe Datenbasis zur Verfügung, um einen DRW festzulegen. Hier kann der DRW für NNH-Untersuchungen von 1500 mGy cm² herangezogen werden. Große Volumina sollten jedoch in der Zahnmedizin eher die Ausnahme sein und müssen durch eine spezielle rechtfertigende Indikation abgedeckt sein.
- *DRW bei Interventionen* : Eine Intervention nach §1 Absatz 8 StrlSchV umfasst sowohl diagnostische als auch therapeutische Zwecke. Wenn der diagnostische Teil unabhängig vom therapeutischen durchgeführt werden kann, so ist das Dosisflächenprodukt ( *DFP* ) dieser Anwendung mit dem jeweiligen DRW zu vergleichen. Beinhaltet die Intervention jedoch neben einem diagnostischen auch einen therapeutischen Anteil, so ist der gesamte Vorgang als therapeutisch zu werten und der entsprechende DRW für interventionelle Eingriffe einzuhalten. Wenn die Dosis bei einer Intervention aufgrund der Komplexität des Eingriffs den entsprechenden DRW voraussichtlich überschreiten wird oder überschritten hat, so ist eine entsprechende Begründung zu dokumentieren, z.B. unter Angabe der Anzahl der betroffenen Gefäße.
- *Altersgruppen in der Pädiatrie* : Die Altersgruppe ab 56 kg (ab ca. 15 Jahre) entspricht der Größe eines schlanken Erwachsenen, sodass für diese Gewichtsklasse keine gesonderten DRW für Jugendliche mehr festgelegt wurden.
- *Planungs-CT in der Strahlentherapie:* Obwohl diese speziellen Anwendungen im Katalog der DRW nicht explizit berücksichtigt sind, sollten zur Vermeidung jeder unnötigen Exposition bei CT-Aufnahmen zur Bestrahlungsplanung („Planungs-CT“) die für die jeweilige Körperregion festgelegten DRW für diagnostische CT-Aufnahmen berücksichtigt werden. Dies ergibt sich aus dem ALARA-Prinzip zur Schonung des gesunden Gewebes, auch wenn die aus der nachfolgenden Strahlentherapie resultierende Exposition im Zielvolumen deutlich höher ist als die des Planungs-CT.
- *Auxiliäre CT-Aufnahmen (Low-Dose-CT) zur Schwächungskorrektur und/oder anatomischen Koregistrierung bei Hybriduntersuchungen an PET/CT- und SPECT/CT-Systemen:* Für diese gelten die DRW aus der Bekanntmachung für nuklearmedizinische Untersuchungen 24 . Werden Aufnahmen in diagnostischer Bildqualität akquiriert, so gelten hingegen die entsprechenden DRW in der jeweils aktuellen Bekanntmachung für Röntgenanwendungen.

## Empfehlungen für einrichtungsspezifische Maßnahmen zur Umsetzung und Erweiterung des Konzepts der DRW

Die in den Bekanntmachungen des BfS festgelegten DRW betreffen häufige und/oder dosisintensive Röntgenanwendungen. Sie enthalten weder konkrete Empfehlungen zur Optimierung der zahlreichen anderen Röntgenanwendungen noch zur praktischen Umsetzung der Betreiberpflichten. Hierzu einige Hinweise:

- *Dosismanagementsysteme* . Es wird nachdrücklich empfohlen, dosisrelevante Größen für alle Röntgengeräte einer Einrichtung mit Hilfe eines Dosismanagementsystems 25 26 27 kontinuierlich automatisiert zu erfassen sowie diese im Rahmen des lokalen Qualitätsmanagements systematisch auszuwerten. Hierbei sollten möglichst auch Angaben zur Körperstatur der Patienten (z.B. Größe, Gewicht, Körperumfang, BMI) berücksichtigt werden, da diese für die Begründung einer Überschreitung eines DRW bei der Überprüfung durch die ÄS oder ZahnÄS relevant sein können.
- *Lokale Referenzwerte.* Trotz der erheblichen Ausweitung des DRW-Katalogs gibt es weiterhin zahlreiche Röntgenanwendungen, für die keine nationalen DRW festgelegt wurden, da sie entweder nicht besonders dosisintensiv sind oder, bundesweit gesehen, nicht sehr häufig zur Anwendung kommen. Werden derartige Untersuchungen in einer Einrichtung jedoch häufiger durchgeführt, so wird empfohlen, lokale DRW anhand des 3. Quartils eines größeren lokalen Patientenkollektivs festzulegen 28 29 30 31 32 . Die Festlegung lokaler DRW bietet sich auch bei Untersuchungsarten an, für die zwar nationale DRW festgelegt sind, diese aber bei Einsatz moderner Geräte und guter Praxis deutlich unterschritten werden können. Diese lokalen DRW könnten sich beispielsweise an den Medianwerten der nationalen Verteilungen der entsprechenden dosisrelevanten Parameter orientieren, die im Supplement zu finden sind.
- *Lokale Qualitätssicherungsteams* . Für die Optimierung des Strahlenschutzes der Patienten – auch über die Einhaltung der DRW hinaus – wird nachdrücklich empfohlen, lokale Teams bestehend u.a. aus Ärzt*innen, Medizinphysiker*innen, IT-Verantworlichen und Medizinischen Technolog*Innen für Radiologie zu etablieren, die regelmäßig die untersuchungsspezifischen Patientenexpositionen analysieren und bewerten. Ziel sollte es sein, Patientenexpositionen auch unterhalb der DRW zu optimieren, um gemäß § 14 Abs. 1 Nr. 5a StrlSchG und § 130 StrlSchV die erforderliche Bildqualität mit möglichst geringer Exposition zu erreichen. Besonderes Augenmerk verdienen hierbei Röntgenanwendungen, die sehr breite Verteilungen der operationellen Dosisparameter (wie z.B. des *DFP* , des *CTDI*_vol_ oder *DLP* ) zeigen.

## Schlussfolgerungen und Perspektiven

Die konsequente Umsetzung des Konzepts der DRW hat sich in der Praxis bewährt und über die Jahre aufgrund konsequenter Protokolloptimierungen und Gerätemodernisierungen zu einer erheblichen Reduktion der Patientenexposition pro Röntgenanwendung gemäß dem ALARA-Prinzip geführt. Diese positive Entwicklung im Sinne des Patientenschutzes rechtfertigt den personellen Aufwand und die ggf. erforderliche Erneuerung von Hard- und Software. Trotz der erzielten Erfolge gibt es weitergehende Überlegungen zur Fortentwicklung sowohl des DRW-Katalogs als auch des grundsätzlichen Konzepts.

Um eine systematische Verzerrung der von den ÄS und ZÄS bei den Betreibern erhobenen Expositionsdaten nach unten zu vermeiden, ist die Vorgehensweise bezüglich der Stichprobenauswahl zumindest so weit zu vereinheitlichen, dass die Auswahl der Röntgenanwendungen nicht den Betreibern überlassen wird, sondern dass seitens der ÄS oder ZÄS konkrete Vorgaben gemacht werden. Nur so kann sichergestellt werden, dass nicht von einigen Betreibern unterdurchschnittlich niedrige Expositionswerte gemeldet werden, die dann in anonymisierter Form für die nächste Aktualisierung der DRW an das BfS weitergeleitet werden.

Für einige klinisch relevante Röntgenanwendungen konnten aufgrund einer fehlenden repräsentativen Datenbasis bislang noch keine DRW festgelegt werden. Dies betrifft beispielsweise CT-Perfusionsuntersuchungen des Gehirns oder Herzens. Zur Orientierung sei diesbezüglich auf die in 33 präsentierten Überlegungen und Expositionsniveaus verwiesen. Ferner hat ein Forschungsvorhaben 34 ergeben, dass DRW für DVT-Anwendungen im Körperstammbereich nicht wie erhofft sinnvoll aus entsprechenden Werten für CT-Untersuchungen abgeleitet werden können, sodass auch diesbezüglich repräsentative Erhebungen erforderlich sind.

Potenziell ist nicht auszuschließen, dass Expositionsparameter zur Einhaltung der DRW betreiberseitig so weit reduziert werden, dass aufgrund veralteter Geräte und Software die Bildqualität für eine zuverlässige Befundung nicht mehr gegeben ist. Das ist derzeit nur zu vermeiden, indem die ÄS oder ZahnÄS die Einhaltung der DRW in Verbindung mit der erzielten Bildqualität bewerten, was nicht durchgängig geschieht. Perspektivisch wäre eine Ergänzung des DRW-Konzepts durch einen Bildqualitäts-Referenzwert vorstellbar, welcher sich auf einen oder mehrere Parameter zur Charakterisierung der Bildqualität bezieht (z.B. das Rauschniveau oder die Detektabilität wohldefinierter Bildmerkmale). Diese Parameter könnten initial nach jeder Untersuchung anhand der akquirierten oder rekonstruierten Bilddaten automatisch berechnet, dokumentiert und mit einer ärztlichen Bewertung der diagnostischen Bildqualität korreliert werden. Erste Versuche in diese Richtung wurden bereits veröffentlicht 35 36 37 38 39 . Konzeptionell bietet es sich an, diese Ansätze mit KI-Techniken des maschinellen Lernens zu kombinieren.
